# Supplementary material for: Manganese Stress Tolerance Depends on Yap1 and Stress-Activated MAP Kinases
Source: Int J Mol Sci. 2022 Dec 11;23(24):15706. doi: 10.3390/ijms232415706 (PMC9779322; doi:10.3390/ijms232415706)
Supplement: Supplementary file 1 [file ijms-23-15706-s001.zip › ijms-2079940-supplementary.pdf]

Supplementary figures:

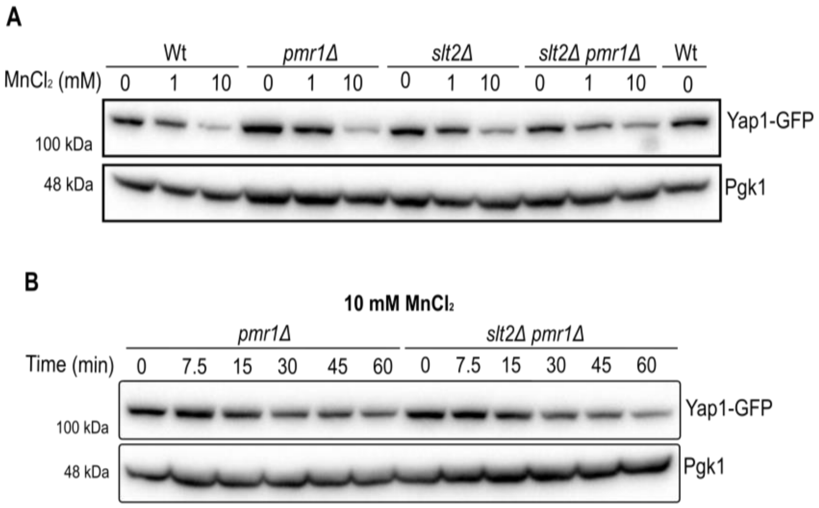

Supplementary Figure S1: MnCl2-driven Yap1 decay is Slt2 independent. **(A)** Analysis of Yap1-GFP protein levels by immunoblotting with anti-GFP antibodies. Strains are indicated. Exponentially growing Wt and indicated mutant strains were treated for 1 h with 1 mM or 10 mM $MnCl_2$ prior to protein isolation. Pgk1 was used as a loading control. **(B)** Comparison of time-dependent Yap1-GFP protein loss in *pmr1Δ* single and *pmr1Δ slt2Δ* double mutants treated with 10 mM $MnCl_2$ for the indicated times (min).
